# IMPORTANCE OF LIVER BIOPSY IN THE DIAGNOSIS OF LYSOSOMAL ACID LIPASE DEFICIENCY: A CASE REPORT

**DOI:** 10.1590/1984-0462/;2018;36;1;00016

**Published:** 2017-10-30

**Authors:** Adriana Maria Alves De Tommaso, Flávia Fonseca de Carvalho Barra, Gabriel Hessel, Carolina Araújo Moreno, Roberto Giugliani, Cecília Amélia Fazzio Escanhoela

**Affiliations:** a Universidade Estadual de Campinas, Campinas, SP, Brasil.; b Universidade Federal do Rio Grande do Sul, Porto Alegre, RS, Brasil.

**Keywords:** Biopsy, Cholesteryl ester storage disease, Fatty liver, Dyslipidemias, Sterol esterase, Biópsia, Doença do armazenamento de éster de colesterol, Fígado gorduroso, Dislipidemias, Esterol esterase

## Abstract

**Objective:**

To describe a case of cholesteryl ester storage disease (CESD) and discuss the importance of liver biopsy for diagnosis.

**Case description:**

A female patient, aged two years and ten months, presented with an increased abdominal volume following hepatomegaly for four months. Abdominal ultrasound demonstrated hepatomegaly and hepatic steatosis. Laboratory tests showed elevated liver serum enzymes and dyslipidemia. Liver biopsy was consistent with CESD.

**Comments:**

Although measuring enzyme activity is the gold standard for CESD diagnosis, liver biopsy is very helpful when investigating suspected cases of CESD, particularly upon other differential diagnoses to be considered.

## RESUMO

**Objetivo:**

Descrever a doença de depósito de ésteres de colesterol (DDEC) e a importância da biópsia hepática na realização do diagnóstico.

**Descrição do caso:**

Paciente feminina, dois anos e dez meses de idade, com queixa de aumento do volume abdominal secundário à hepatomegalia há quatro meses. Ultrassonografia abdominal demonstrou hepatomegalia e esteatose hepática. Exames laboratoriais mostraram aumento de enzimas hepáticas e dislipidemia. A biópsia hepática foi compatível com DDEC.

**Comentários:**

Embora a medida da atividade enzimática seja o padrão-ouro para o diagnóstico de DDEC, a biópsia hepática é muito útil na investigação de casos suspeitos, particularmente quando há outros diagnósticos diferenciais a serem considerados.

## INTRODUCTION

Wolman Disease and cholesteryl ester storage disease (CESD) are rare entities caused by complete or partial lysosomal acid lipase (LAL) deficiency, which is encoded by the LIPA gene located at 10q23.31. This enzyme mediates the intracellular hydrolysis of cholesteryl esters and triglycerides.^1^ ^,^ ^2^

In Wolman Disease, there is total enzyme deficiency, characterized by abdominal distension, hepatosplenomegaly, ascites, intestinal malabsorption with diarrhea, adrenal calcification, and failure to thrive. The disease progresses rapidly, causing death before the first year of life. In CESD, enzyme deficiency is partial and signs and symptoms vary widely. Hepatosplenomegaly, elevated serum transaminase levels, hyperlipidemia, hypercholesterolemia, low serum levels of high-density lipoprotein cholesterol (HDL), vomiting, diarrhea, growth failure, and liver steatosis can occur.^1^ ^,^ ^3^

Fatty liver in childhood develops for several reasons,^4^ and CESD has a broad clinical and laboratory profile.^1^ ^,^ ^3^ ^,^ ^5^ The aim of this paper is to describe the first case of CESD identified at UNICAMP Clinics Hospital and the importance of liver biopsy for diagnosis.

## CASE REPORT

Female patient, aged 2 years and 10 months, presented with complaints of enlarged abdominal volume for four months. At physical examination, she weighed 12.3 kg (P3-P10), and was 86-cm height (P25), with body mass index (BMI) of 16.8. The liver was palpable approximately 6 cm below the right costal margin, without splenomegaly. The abdominal ultrasonography demonstrated hepatomegaly and liver steatosis. Laboratory tests showed: ALT=57 U/L (normal value: up to 39); AST=65 U/L (normal value: up to 56); cholesterol=373 mg/dL (normal value: up to 170); HDL=19 mg/dL (normal value >40); and triglycerides=217 mg/dL (normal value up to 100). Glucose, albumin, INR values, and complete CBC were normal.

**Figure 1:**
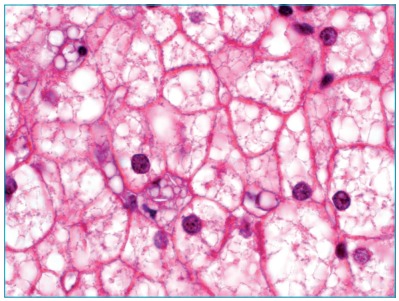
Intense microvesicular steatosis with focal deposition of cholesterol crystals in Kupffer cell at optical mycroscopy.

The clinical hypothesis was glycogen storage disease (GSD) with good fasting tolerance. Liver biopsy was indicated and showed diffuse enlargement of hepatocyte volume with intense microvesicular steatosis and ballooning, as well as triglyceride deposits in Kupffer cells and portal macrophages, associated with occasional deposition of cholesterol crystals (Figure 1). Histologic findings were suggestive of Wolman Disease/CESD. Adrenal calcification was not present. Liver function was preserved and the patient’s general health was good. Thus, if LAL deficiency existed, clinical presentation would be consistent with CESD.

A sample sent to laboratory analysis of acid lipase activity in leukocytes tested 2.8 nmol/h/mg (reference value: 112-378), which is consistent with biochemical diagnosis of LAL deficiency. LAL activity was measured according to criteria suggested by Civallero et al.^6^ at the Laboratory of Inborn Errors of Metabolism, Medical Genetics Service, Hospital de Clínicas de Porto Alegre-the main reference center for diagnosis of lysosomal diseases in Brazil.

**Figure 2:**
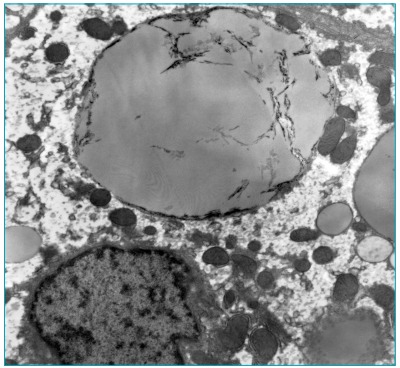
Triglyceride deposits were sometimes fragmented with irregular striations. (“bitten-apple” aspect).

Upon electron microscopy, we noted massively distended hepatocytes due to large amount of round triglyceride deposits, varying in size and lysosomes or lying freely in cytoplasm. These cells were sometimes fragmented with irregular striations, likely corresponding to the removal of cholesteryl esters. Needle-shaped spaces were overlapping triglyceride vacuoles (“bitten apple” aspect). There were also triglyceride deposits in cytoplasm of macrophages (portal and Kupffer cells). The ultrastructural findings were consistent with the previous morphological hypothesis of Wolman Disease/CESD (Figure 2). The patient’s parents signed an informed consent form allowing the publication of this case.

## DISCUSSION

Diagnosis of CESD is challenging, for it shares clinical and laboratory characteristics with conditions such as nonalcoholic fatty liver disease, familial combined hyperlipidemia, and several metabolic disorders.^4^ ^,^ ^5^ ^,^ ^7^ In our case, the initial hypothesis was hepatic GSD (incidence of approximately 1/30,000), condition that is more common than CESD (~1/130,000).^8^ ^,^ ^9^ In both conditions, hepatomegaly and hyperlipidemia can progress with no significant changes in neuromotor development or without being notable at physical examination. However, a routine histological examination was important to distinguish it. In GSD, hepatocytes become turgescent due to glycogen accumulation, as shown by periodic acid Schiff (PAS) staining and the large lipid vacuoles eventually found in cytoplasm. Glycogen displaces mitochondria and other cytoplasmic organelles toward the cell membrane, resulting in the appearance of a pseudothickening, plant-like hepatocyte membrane.^10^

The following histological signs have been described in CESD: predominantly microvesicular steatosis involving hepatocytes, Kupffer cells, and portal macrophages, with progression to fibrosis and micronodular cirrhosis.^11^ Hůlková and Elleder^12^ evaluated liver biopsy specimens from 19 patients with CESD to determine the histopathological criteria that distinguishes this condition from other forms of microvesicular steatosis, such as nonalcoholic fatty liver disease and other nonlysosomal metabolic conditions. They reported a histochemical test with cathepsin D readily able to identify accumulation of lipids in lysosomes, facilitating the diagnosis of CESD. Furthermore, pathognomonic birefringent cholesteryl crystals were seen in fresh tissue under polarized light.

In this case report, clear needle-shaped spaces observed in Kupffer cells and portal macrophages by optic microscopy, corresponding to cholesterol crystals that were eliminated during histological processing, guided the diagnosis. By electron microscopy, lipid deposits in the form of triglycerides in Kupffer cells and portal macrophages, associated with storage of cholesterol crystals in hepatocytes, was considered sufficient for the diagnosis of Wolman Disease/CESD.

Enzyme replacement therapy has been used effectively in clinical trials,^13^ and has already been approved in European Union and USA since 2015, Although it still pends approval in Brazil. Thus, early diagnosis of this morbid condition is critical. Histopathological alterations can be suggestive of this disease and lead to conclusive testing. Although measuring enzyme activity is the preferred method for diagnosing CESD, liver biopsy is fundamental when suspecting it, particularly when other differential diagnoses are considered.
